# Think about the joints

**DOI:** 10.1186/1546-0096-9-S1-P116

**Published:** 2011-09-14

**Authors:** ET Mosley, AM McMahon

**Affiliations:** 1 Sheffield Children’s Hospital, Sheffield, UK; 2 Leeds Children’s Hospital, Leeds, UK

## Background

Musculoskeletal problems are common in children. The majority are self-limiting and related to trauma; however the symptoms can be feature of serious medical conditions.

It has been previously shown that musculoskeletal examination is poorly documented. Musculoskeletal examination is a station on the RCPCH clinical exam. Teaching resources on how to perform musculoskeletal examinations are freely available together with guidelines on when musculoskeletal examination should be performed.

## Aim

Recent research has shown that such examinations are highly acceptable to parents and patients; therefore we aimed to see if the situation has improved.

## Method

Random selections of patients attending the assessment unit at a large tertiary children hospital in a 1-week period were reviewed. The inclusion of a musculoskeletal examination was reviewed.

Standard used for inclusion of musculoskeletal examination:

• Child with muscle joint or limb pain

• Unwell child with pyrexia

• Limping child

• Delay of milestone

• Clumsy child in the absence of neurological signs

• Associated conditions/chronic diseases e.g. inflammatory bowel, cystic fibrosis, arthritis, psoriasis

Red Flags (concern about infection, malignancy or NAI)

• Fever, malaise

• Bone/joint pain

• Refractory pain, persistent night sweats

• In congruency between history and presentations

## Results

20 admissions, totalling 49 patients were reviewed.

20 patients had presenting symptoms to advocate the inclusion of musculoskeletal examinations

**Table:** 

Trigger	Number
Fever	17
Limp/joint pain	1
Red flag (excluding fever)	2

Only 2 patients had documented musculoskeletal examinations.

## Conclusions

Despite the availability of educational training resources there has been little improvement in the inclusion of this musculoskeletal examination in clinical practice.

Whilst this was a small sample it highlights the need for further education at all levels in when and how to perform a musculoskeletal examination.

